# Lung Transplantation in a Patient With COVID-19-Associated Acute Respiratory Failure

**DOI:** 10.7759/cureus.17152

**Published:** 2021-08-13

**Authors:** Fatima Sajid, Taha Ahmed, Maher A Baz, Michael I Anstead

**Affiliations:** 1 Pulmonology, University of Kentucky, Lexington, USA; 2 Hospital Medicine, University of Kentucky, Lexington, USA

**Keywords:** lung transplant, covid-19, acute lung injury, lung fibrosis, respiratory failure

## Abstract

Coronavirus disease 2019 (COVID-19) is currently a significant cause of acute respiratory failure worldwide, leading to irreversible fibrotic lung disease. In patients with persistent respiratory failure after acute COVID-19 infection, lung transplant is an emerging option. Here, we have presented a case where the patient required venovenous extracorporeal membrane oxygenation (VV-ECMO) support for 33 days until a bilateral lung transplant was performed on day 71 after the initial COVID-19 infection. The early outcomes have been favorable. Currently, no guidelines exist for an acceptable time period after initial COVID-19 infection, duration of negative COVID polymerase chain reaction (PCR) testing, or negative Vero cell culture in the setting of persistent positive COVID PCR testing before listing for a lung transplant. Due to a lack of standardized guidelines, this patient was not listed for a lung transplant until the COVID-19 PCRs came negative on days 47 and 49 after the infection.

## Introduction

COVID-19 is a significant cause of acute respiratory failure worldwide. In patients with persistent respiratory failure after acute COVID-19 infection, lung transplant is an emerging and life-saving option. Here, we have presented a case of bilateral lung transplantation in a patient with COVID-19-associated irreversible respiratory failure.

## Case presentation

On January 25, 2021, a 43-year-old man tested positive for COVID-19, and eight days later, he was admitted to a community hospital due to worsening shortness of breath and hypoxia. He had a medical history of ankylosing spondylitis, hypertension, gastroesophageal reflux disease, and hyperlipidemia and was previously treated with adalimumab for more than one year before the illness. After admission, he received treatment with one dose of ivermectin and one dose of tocilizumab (second-day postadmission), azithromycin and remdesivir (second- to fifth-day postadmission); and Zosyn, linezolid, and dexamethasone (from third-day postadmission). According to the Infectious Diseases Society of America (IDSA) guidelines, tocilizumab should be added to the standard of care (steroids) for severe or critical COVID-19 patients. The hospital stay was complicated by bilateral deep vein thrombosis (DVT) diagnosed on day 14. He was treated with enoxaparin and later switched to rivaroxaban for DVT. Due to worsening hypoxemia, he was escalated to 100% fraction of inspired oxygen (FiO2) through a high-flow nasal cannula (HFNC) and intermittent bilevel positive airway pressure (BiPAP) on day 31.

**Figure 1:**
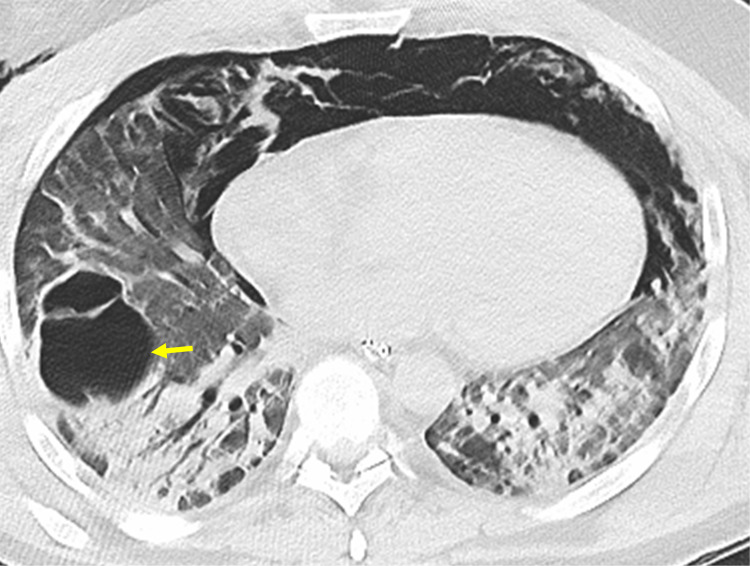
CT scan of the chest without contrast CT scan of the chest without contrast (axial image) shows extensive pneumomediastinum and small right pneumothorax. Pneumatocele (yellow arrow), mild bronchiectasis, ground-glass opacities, and consolidations in bilateral lower lobes can be seen.

On day 32, he was transferred to our tertiary care center and was intubated and placed on the ventilator. Bronchoscopy was performed that showed diffuse alveolar hemorrhage. Anticoagulation was discontinued because of its potential to cause bleeding. On day 33, an inferior vena cava (IVC) filter was placed. Therapeutic paralysis was induced from days 33 to 35, and prone positioning was twice utilized with no improvement in oxygen saturation. CT chest performed on day 37 showed pneumatocele in the right lower lobe, significant air space, and bilateral interstitial disease in lower lobes (Figure 1).

**Figure 2:**
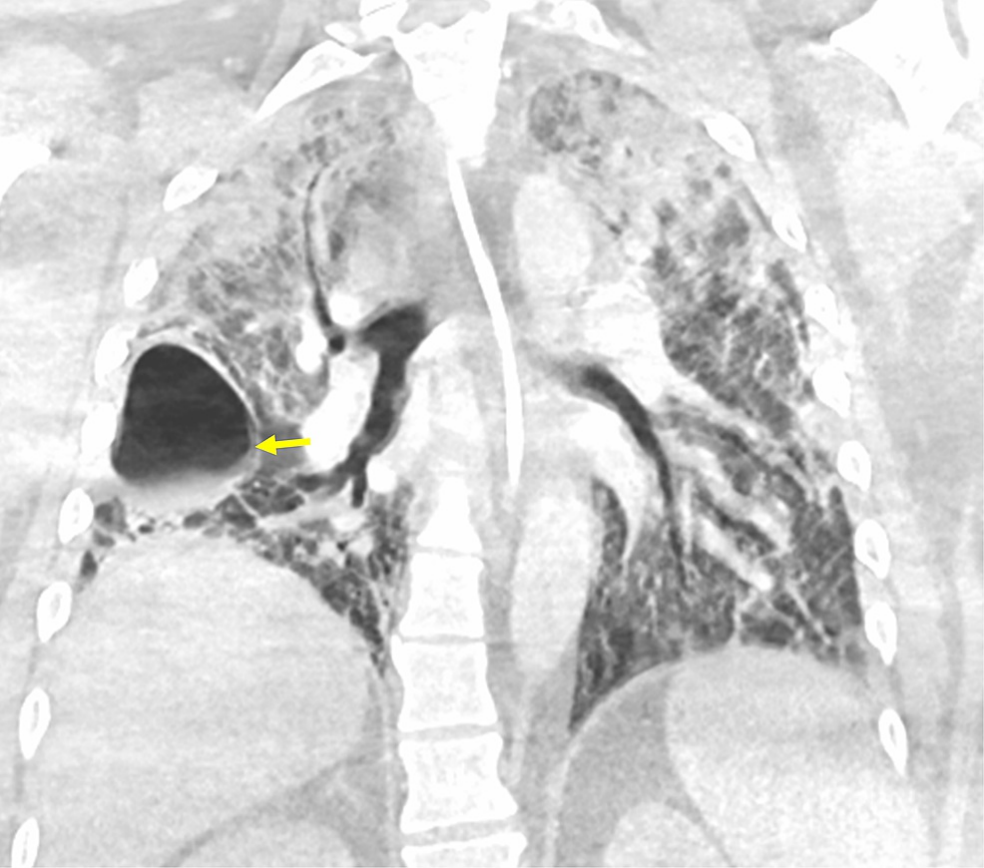
CT scan of the chest with contrast (coronal image) shows bronchiectasis involving predominantly lower lobes, reticular interstitial opacities, pneumatocele (yellow arrow), and upper lobe airspace opacities

**Figure 3:**
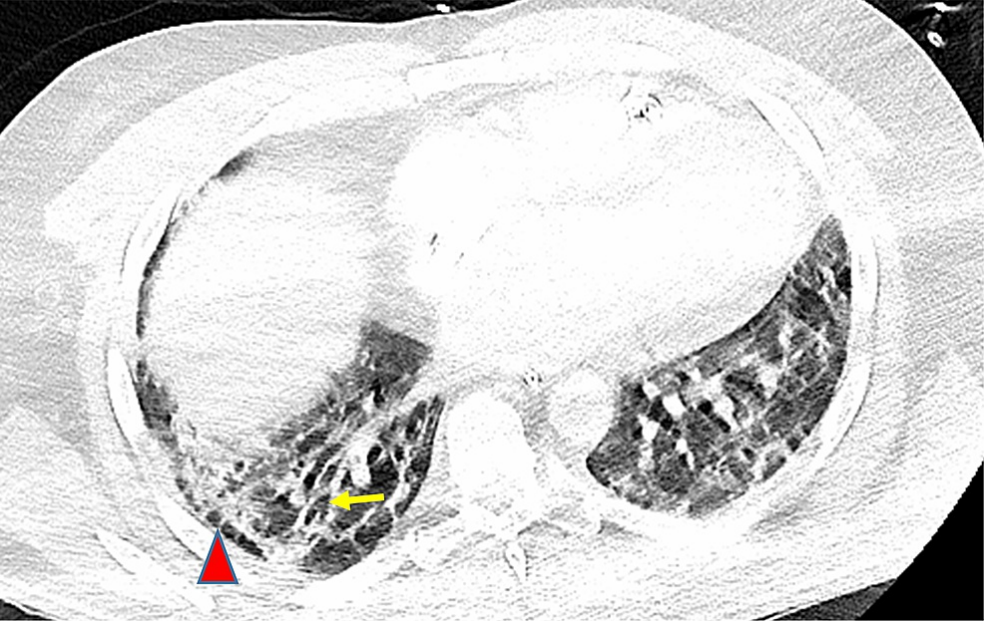
CT scan of the chest with contrast (axial image) shows traction bronchiectasis (yellow arrow) and subpleural reticulations (red arrowhead) in the right lung base that is suggestive of progressive fibrosis

Due to the ongoing poor oxygenation and ventilator-associated barotrauma on day 39, he was placed on venovenous extracorporeal membrane oxygenation (VV-ECMO) and was extubated to prevent further ventilator-induced lung injury. COVID-19 polymerase chain reaction (PCR) test was repeated on day 40, and it was positive. Subsequent COVID-19 PCR tests performed on days 47 and 49 came out negative. On day 52, he was listed for a bilateral lung transplant based on the severity, irreversibility of the respiratory failure, and at least 28 days of negative COVID-19 PCR results since the onset of severe lung injury. His lung allocation score (LAS) was 2.77. CT chest performed on day 50 showed progressed bilateral postinflammatory lung fibrosis with persistent pneumatocele (Figures 2, 3). He continued to require 100% HFNC and ECMO circuit at 100% along with a 100% non-rebreather mask as needed to keep his oxygen saturation 94%-98% at rest. His human leukocyte antigen (HLA) antibody screen was negative. On day 71, he underwent a bilateral lung transplant and was intubated in the operating room. The ECMO circuit was removed on day 72, and he was extubated on day 74.

**Figure 4:**
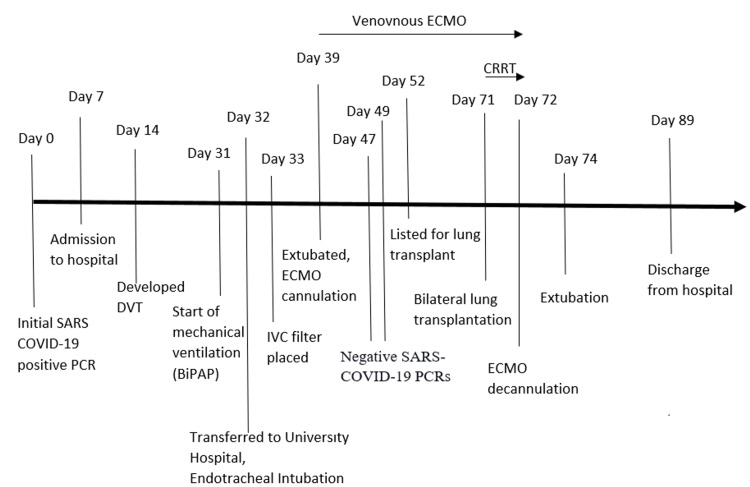
Timeline of clinical events BiPAP: bilevel positive airway pressure, CRRT: continuous renal replacement therapy, DVT: deep vein thrombosis, ECMO: extracorporeal membrane oxygenation, IVC: inferior vena cava, PCR: polymerase chain reaction, SARS: severe acute respiratory syndrome.

Postsurgery, he was given pulse steroids (40 mg, 30 mg, and 20 mg each once a day), tacrolimus 2 mg every 12 hours, and mycophenolate mofetil 1000 mg every 12 hours for immunosuppression. Mycophenolate mofetil was switched to azathioprine due to diarrhea. Posttransplant, he developed septic shock due to donor-associated pneumonia and acute kidney injury (AKI). He was treated with vasopressin, epinephrine, norepinephrine, IV meropenem, IV ceftaroline, and IV micafungin. Continuous renal replacement therapy (CRRT) was done through a tunneled catheter from days 72 to 74 (2 to 4 days posttransplant) until his AKI improved. He was extubated on day 74 and was able to speak. Chest X-ray done on day 74 showed an absence of any new focal consolidation, significant pneumothorax, or large pleural effusion. However, a small apical pneumothorax was seen. Bronchoscopy performed on day 75 showed an absence of purulent secretions, and the donor cultures were negative for any growth. HFNC was weaned to a 6 L nasal cannula on day 76. On day 88, bronchoscopy with bronchoalveolar lavage (BAL) and biopsy was done. The biopsy showed two small foci of minimal acute rejection and was graded A1B0. On day 89, he was discharged home on room air with oxygen saturation of 99% and walking 600 feet on his own. The renal function had recovered to normal, and a right chest tube remained due to persistent pleural drainage. For immunosuppression, he was prescribed tacrolimus 3 mg twice a day, prednisone 20 mg daily, and azathioprine 200 mg once a day. The timeline of the patient is shown in Figure 4.

## Discussion

More than 72 million cases and 1.6 million deaths worldwide have been caused by the COVID-19 pandemic as of December 2020 [1]. A significant number of people develop severe symptoms leading to acute respiratory distress syndrome (ARDS) ultimately requiring mechanical ventilation and prolonged intensive care unit (ICU) [2]. Lung transplantation is a life-saving option for the treatment of COVID-19-associated irreversible respiratory failure. However, it is performed rarely due to the infectious etiology behind acute lung injury. There is a concern of severe acute respiratory syndrome coronavirus 2 (SARS-CoV-2) or any superinfecting pathogen recurring in the allograft [3]. Moreover, many patients with acute respiratory failure due to COVID-19 have other comorbidities that preclude them from receiving a transplant including renal dysfunction, muscle wasting, and other organ failures. Although the early outcomes after lung transplant have been favorable, there is a high chance of publication bias in the existing literature as the cases with poor outcomes are not published [4]. 

Only a limited number of people with COVID-19-associated respiratory failure are considered for a transplant. Positive outcomes are expected to be seen in patients who are younger, have only a single organ failure and adequate body mass index, are awake to discuss the transplantation, can do physical rehabilitation while waiting on the list, and have negative COVID-19 PCR results [4]. A study has shown that the risk of mortality is higher in patients who undergo surgery with positive COVID-19 PCR. This is a matched cohort study including 123 patients (41 with COVID-19 [33.3%] and 82 controls [66.7%]) [5]. In this case, the patient was not listed until COVID-19 PCR was finally repeatedly negative after day 47. In another case report, Vero cell culture for COVID-19 was confirmed negative before listing a patient with repeatedly positive COVID PCR testing [6]. Currently, no guidelines exist for an acceptable time period after initial COVID-19 infection, duration of negative COVID PCR testing, or negative Vero cell culture in the setting of persistent positive COVID PCR testing before listing for a lung transplant. Hence, more work needs to be done.

The patient mentioned here required VV-ECMO support for 33 days until a bilateral lung transplant was performed on day 71 after the initial COVID infection. The postoperative survival rate after lung transplantation despite being on ECMO has been reported to be as high as 95% [1]. On the other hand, the 90-day in-hospital mortality after the initiation of ECMO in patients with ARDS has been reported to be 38% (95% CI, 34.6-41.5) [7]. Hence, lung transplantation offers better outcomes in patients with ARDS who are on ECMO support.

## Conclusions

A significant number of people with COVID-19 develop acute respiratory distress syndrome, leading to irreversible fibrotic lung disease. The majority of them require ECMO support and have a poor prognosis. Lung transplantation becomes a life-saving option in such cases. A favorable early outcome has been seen in this patient with COVID-19-associated irreversible respiratory failure and pulmonary fibrosis.
